# Climate change, disaster management and primary health care in Zimbabwe

**DOI:** 10.4102/phcfm.v14i1.3684

**Published:** 2022-09-30

**Authors:** Sunanda Ray, Tinashe Goronga, Phillip T. Chigiya, Farai D. Madzimbamuto

**Affiliations:** 1 Department of Medical Education, Faculty of Medicine, University of Botswana, Gaborone, Botswana; 2 Centre for Health Equity, Harare, Zimbabwe; 3 Department of Anaesthetics and Critical Care, Faculty of Medicine, University of Botswana, Gaborone, Botswana

**Keywords:** climate change, primary health care, cyclone, Cyclone Idai, Zimbawe

## Abstract

The health crises related to climate change in African countries are predicted to get worse and more prevalent. The response to catastrophic events such as cyclones, flooding and landslides must be rapid and well-coordinated. Slower adverse events such as droughts, heat stress and food insecurity must similarly be anticipated, planned for and resourced. There are lessons to be learnt by the health system following the crisis created by Cyclone Idai in Zimbabwe during March 2019, which required a massive humanitarian response to mitigate the impact of torrential rainfall on lives and livelihoods. Several researchers and organisations documented the emergency response in detail. They reported that the government response was hampered by a lack of preparedness, poor planning, inadequate resource mobilisation and weak coordination. Rural communities did not access the early warning cyclone alerts disseminated through television, print and social media, nor did they appreciate the seriousness of events until it was too late. Primary health care (PHC) teams are familiar and trusted by the communities they serve and have a critical role in raising public awareness and in documenting the evolving impact of climate change, using established health indicators and local narratives. PHC leaders and providers have the knowledge and skills to mediate between government bodies, international agencies, other stakeholders and communities on the predicted impact of climate change on health outcomes, highlighting the vulnerability of disadvantaged and impoverished groups. They are also able to work with community leaders, using indigenous knowledge on weather patterns, to build local engagement in protection plans.

**Contribution:**

This article describes the role health professionals and civil society can play in educating the public on the dangers faced in the near future as a result of climate change and actions that can be taken to become more resilient and to mitigate this impact.

## The role of primary health care in disaster management

Primary care providers (PCPs) have a crucial role in the urgent response needed to address the impact of climate change on communities, especially the most vulnerable and disadvantaged.^1,2,3^ Because of their proximity to the people they serve, they are trusted as planners and coordinators of healthcare, leaders and advocates, with a special responsibility in raising public awareness and calls to action in emergency responses to disasters.^1^ Like many African countries, climate change in Zimbabwe is forecast to increase the future occurrence, intensity and magnitude of floods, droughts, heat stress and disease outbreaks, with corresponding social and economic disruption and millions of people affected. Key economic sectors of agriculture, water, energy, forestry, tourism and industry will also be impacted by climate variability.^4^ The poor, disadvantaged and marginalised are 50% more likely to bear the impact of climate change than non-poor people, with more pushed into poverty as a result of adverse climate events.^4^

Zimbabwe’s multisectoral and interdisciplinary disaster risk management (DRM) system is led by a National Civil Protection Committee with coordination from the Ministry of Local Government, Public Works and National Housing. This ministry relies heavily on international agencies for funding and implementation of emergency responses.^5,6,7^ So, when disasters occur, health service leaders and PCPs are often bypassed or marginalised in the arrangements.^1^ To explore the potential role of primary health care (PHC) in DRM, the case study of the health crisis created by Cyclone Idai in Zimbabwe is briefly presented here.

## Cyclone Idai

The impact of Cyclone Idai in Manicaland province (Eastern border of Zimbabwe) in March 2019 has been documented in detail because it caused extensive damage to homes, agriculture, roads, bridges, communications, electricity supplies, schools and health centres, together with disruption to livelihoods, food security and loss of life.^5,6,7,8,9,10,11,12,13^ At least 270 000 people were affected (129 600 children), with more than 340 dead, 51 000 displaced and many missing in Zimbabwe because of torrential rainfall, severe flooding and mudslides.^10^ The 1500 km of roads that were damaged prevented access to essential services immediately and for several months following the cyclone.^5,9,12^ The water supply network in Chipinge district was disrupted, leaving more than 30 000 people without access to safe drinking water, who were instead supplied by privately funded water truck deliveries.^7^ Manicaland health facilities initially recorded 354 cyclone-related hospital admissions, which escalated to 21 952 by May, of whom 64.3% were women.^5^ Acute respiratory infections, malaria, dysentery and diarrhoea cases were reported by health facilities, with active surveillance conducted for cholera and typhoid.^10,11^ Trauma, injuries, multiple fractures and lacerations constituted the main immediate health repercussions, with women, children and the elderly as the most vulnerable. Psychological and post-traumatic stress was reported for health staff working with survivors as well as amongst those who had witnessed or suffered personal loss resulting from the cyclone.^6,9^

The immediate outpouring of humanitarian support for the two districts most affected was significantly from local civil society groups, non-governmental organisations (NGOs), churches, private individuals and international organisations, providing food relief, clothes, water supplies and medications.^6,7,12,13^ Emergency food supplies were provided to approximately 133 200 individuals.^7^ At least 300 internally displaced persons were given refuge inside a hotel in one of the affected towns while others were sheltered in tents.^7,8^ Casualties were initially treated in trauma units and first aid tents in open fields, then later air-lifted to hospitals for surgical management.^9,12,13^ Volunteers from medical associations travelled from the capital city to the district hospitals to replenish supplies and carry out surgery.^12,13^ Health facilities received emergency medical supplies and 11 satellite clinics were established to provide emergency health services. Many of those who needed help had chronic conditions, diabetes, hypertension, asthma and HIV, who had lost their medications along with their homes. People living with HIV usually receive 3-months’ supply at one time so the ones worst affected were those who lost their medication when their homes were destroyed.^1,9,12^ Proactive immunisation against cholera and measles was carried out while counselling centres provided some limited psychosocial, mental health and child protection support at a few clinics in the two districts.^5,8,10^ New boreholes were drilled to resuscitate water sources; water purification tablets and hygiene materials were distributed to reduce the risk of water-borne diseases such as cholera.^7,8^ Disaster risk reduction (DRR) training and emergency drills were conducted for district civil protection committees (CPCs), targeting government employees, school teachers and pupils, humanitarian agencies and NGOs.^8^ Crop seed and farming equipment were provided to resuscitate farming livelihoods, although only a few households received this assistance.^8^

Studies conducted in the aftermath of the cyclone found that the government response was retarded by fragmented disaster planning, inadequate resource mobilisation, inexperienced personnel on the ground and dependence on donor funding, followed by inadequate reconstruction and rehabilitation follow-up measures.^5,6,8^ Local government bodies who were meant to lead the emergency response were unprepared and not visible at the onset of the disaster. Coordination of the various agencies providing humanitarian assistance, civil and social protection was problematic. There were capacity, regulation and policy shortcomings, poor information-sharing, duplication of interventions on the one hand and neglect of essential health needs on the other.^6^ The tents provided by the United Nations High Commissioner for Refugees (UNHCR) as temporary accommodation were still providing shelter two years later because recovery strategies failed to include permanent housing in that time.^8^ District CPCs and communities had limited awareness of disaster vulnerability and risks associated with flood hazards so they failed to anticipate the seriousness of the early warning alerts notified through various forms of media (television, print and WhatsApp), which are not readily accessed by rural communities.^8^

## The role of primary health care in addressing climate change

The build-back-better approach to post-disaster recovery presents opportunities to create safer, sustainable and more resilient communities as part of reconstruction and rehabilitation.^8^ Primary care providers can contribute not only to rebuilding programmes and primary care services disrupted by adverse climate events such as Cyclone Idai but also in long-term protection efforts. The principles underlying PHC include social determinants of health, including human rights to safe water and sanitation, shelter, food security and access to healthcare, so encompass a range of interventions that underpin the build-back-better approach. This requires a multidisciplinary and intersectoral team approaches utilising the skills of all PHC providers and partners in the health system and the community, especially village health workers and environmental health assistants. Building-back-better would mean PHC working with public health colleagues to improve surveillance of communicable and non-communicable diseases in relation to climate change and monitoring trends with regard to ambient temperature, air pollution, rainfall, drought and seasons. A gap currently in understanding health impact is data linking routine health information with environmental conditions. The PHC role also extends to strengthening communities to be able to protect themselves and be less vulnerable from future adverse climate events.^2^ Leadership from PHC, including through academic colleges and professional associations, has a major role to play in this through advocacy and coordination, to influence policy and research priorities and to emphasise the urgency and gravity of the climate change and health nexus.^1,2,3,14,15^

Public education and awareness on climate change and health are key PHC team activities.^3,15^ These need to be ongoing, persistent and persuasive and build on the DRR training conducted with the CPCs. Educating communities, for example, about the relationship between deforestation, rainfall, mudslides and soil erosion would be more effective if alternatives to wood and charcoal are made cheaper and easily available. The relationship between household air pollution and childhood respiratory complaints and burns could be explained to motivate the use of alternative cooking fuels.^16^ These are some multisectoral and community-empowerment PHC-based solutions to climate change-related problems and mitigation efforts. Research has identified that some community members in Zimbabwe study weather patterns, trees and clouds, alongside the behaviour of particular birds and animals, to predict imminent flooding and other adverse climate events. Communities take preventive action (e.g. moving to higher ground or building temporary bridges) because they trust these indigenous knowledge systems.^17^ Partnering with them as part of the PHC and DRR approaches to climate-related stresses could improve communities’ engagement in actions required to respond to increased ambient temperatures, air pollution, veld fires as well as with early warning alerts for torrential rain and flooding. Primary health care leaders and district CPCs can also work together to ensure minimal disruption and restoration of supply chains to ensure effective responses and adequate preparation for climate change-related emergencies.^2^

In conclusion, the health sector must negotiate to be more directly involved in decision-making, planning and policy development about the impact of climate change on the health of communities. Primary care providers are well-placed to mediate between government bodies, international agencies, other stakeholders and communities, so they should be recognised as central to leadership, advocacy and public health protection in the climate change and health nexus.
